# A Laboratory Experimental Study on Enhancing the Oil Recovery Mechanisms of Polymeric Surfactants

**DOI:** 10.3390/molecules29061321

**Published:** 2024-03-15

**Authors:** Junhui Guo, Fulin Wang, Yunfei Zhao, Peng Wang, Tianzhi Wang, Jixiang Yang, Bo Yang, Liangliang Ma

**Affiliations:** 1 MOE Key Laboratory of Enhanced Oil Recovery, Northeast Petroleum University, Daqing 163712, China; guojunhui@petrochina.com.cn; 2 E&D Research Institute of PetroChina Daqing Oilfield Co., Ltd., Daqing 163712, China; wangtianzhi@petrochina.com.cn (T.W.); yangjixiang@petrochina.com.cn (J.Y.); 3 PetroChina Daqing Oilfield Co., Ltd., Daqing 163002, China; zhaoyunfei@petrochina.com.cn (Y.Z.); wangpeng1@petrochina.com.cn (P.W.); mal031@petrochina.com.cn (L.M.); 4 Test Technical Services Branch of Daqing Oilfield Company Ltd., Daqing 163712, China; dlts_yangb@petrochina.com.cn

**Keywords:** polymeric surfactant, EOR mechanism, onshore multi-layered sandstone reservoir

## Abstract

In order to evaluate the physical and chemical properties of polymer surfactants and analyze their oil displacement mechanisms, three types of poly-surfactant used in the Daqing oil field were chosen to be researched, and the oil displacement effects were studied using poly-surfactants of different viscosity, dehydrating rate, and core permeability. The main purpose is to determine the reasonable range of different characteristic indexes of polymeric surfactant flooding. The oil displacement effect of 15 cores was analyzed, and the effects of viscosity, the dehydrating rate of emulsion, and permeability on EOR (Enhanced Oil Recovery) were analyzed. The oil displacement mechanisms of polymeric surfactants were researched using a photolithographic glass core. This paper explores the mechanism underlying production enhancement as an EOR target, while simultaneously conducting laboratory tests to assess the physical and chemical properties of polymeric surfactants. The poly-surfactant agents exhibit a notable increase in viscosity, with the optimal displacement effect observed at a core effective permeability exceeding 400 mD, resulting in a potential EOR of 15% or higher. Moreover, at a viscosity ranging between 40 and 70 mPa·s, the total EOR can reach 73%, with the peak efficiency occurring at a viscosity of 60 mPa·s. The water loss rate of the emulsion, ranging between 30% and 70%, achieves optimal performance at 50%. The poly-surfactants’ higher viscosity extends the oil sweep area, enhancing recovery efficiency, and noticeably reducing residual oil compared to water flooding. During poly-surfactant flooding, a substantial amount of residual oil is extracted and transformed into droplets. The rapid emulsification of the polymeric surfactant solution with crude oil forms a stable emulsion, contributing to its significant oil recovery effect. This research provides valuable technical support for EOR in thin and low-quality reservoirs of onshore multi-layered sandstone reservoirs.

## 1. Introduction

The remaining reserves in older oil fields are substantial, and these fields are expected to continue being the primary drivers of oil field development in the foreseeable future [1,2]. Chemical flooding has emerged as a common technique for old oil fields, yielding positive development outcomes. Key methods include polymer flooding [3,4], alkali/surfactant/polymer (ASP) flooding [5,6], binary flooding [7], and deep profile control, incorporating particles [8], gel [9], and asphalt [10,11,12], among others. In Daqing oil field application tests, polymer flooding showed an EOR of 8–10%, while ASP flooding exhibited an approximately 20% improvement [5,6]. However, polymer flooding faces challenges such as inadequate salt resistance and shear resistance. In practical oil field applications, polymer injection from the wellhead to the bottom of the well can lead to a viscosity reduction from 120 mPa·s to 30–40 mPa·s, with a viscosity reduction rate of nearly 70%. Moreover, the polymer’s profile control and displacement capabilities may not suit various types of reservoirs. ASP flooding, on the other hand, presents difficulties in oil field management, particularly with scaling issues that can cause wellbore blockages, rendering production impossible. This situation significantly increases the need for cleaning, resulting in the waste of manpower, material resources, and financial means [7,13,14]. As a novel oil flooding agent, poly-surfactants offer advantages such as reversible aggregation and lower molecular weight. They possess characteristics such as anti-biodegradation, anti-oxidation, anti-mechanical shear, temperature and salt resistance, high viscosity, good solubility, and a fast dissolution rate [15]. Suitable for various permeability reservoirs, they can adapt to different-salinity oil reservoirs and be injected directly with an effluent. They represent an effective chemical drive that is more economical and environmentally friendly and hold promising prospects for oil development. As a new EOR technology following previous polymer flooding, poly-surfactant agents combine flow control and emulsifiability, enhancing oil flooding efficiency and expanding the oil sweep volume [16,17,18,19,20]. Pilot tests in the Daqing oil field involving polymeric surfactant flooding were conducted, yielding positive results. However, the understanding of the EOR mechanism is not yet comprehensive. To further apply this new type of surfactant to field experiments and applications, new laboratory tests were conducted to evaluate its oil displacement effect. In the meantime, the physical and chemical properties of polymeric surfactants were examined in the laboratory. The poly-surfactant agent, with its higher viscosity-increasing property, expands the oil sweep area, offering robust technical support for oil field development.

## 2. Results and Discussion

This investigation focused on the properties of poly-surfactant agents’ oil displacement effects based on different poly-surfactant viscosities, adsorption properties, water evolution rates, concentrations, and core permeabilities. The primary objective was to determine the reasonable range of different characteristic indexes for polymeric surfactant flooding.

### 2.1. Physical and Chemical Properties of Poly-Surfactant

#### 2.1.1. High Viscosity Improvement Ability

The viscosity of 1000 mg/L of the poly-surfactant solutions was continuously measured using a Brookfield viscometer at 6 RPM and 45 °C for 300 s. The results indicated the existence of a concentration threshold, beyond which the contact between molecules increased, enhancing the self-cross-linking ability among molecules.

#### 2.1.2. Shear Dilution

**Figure 1:**
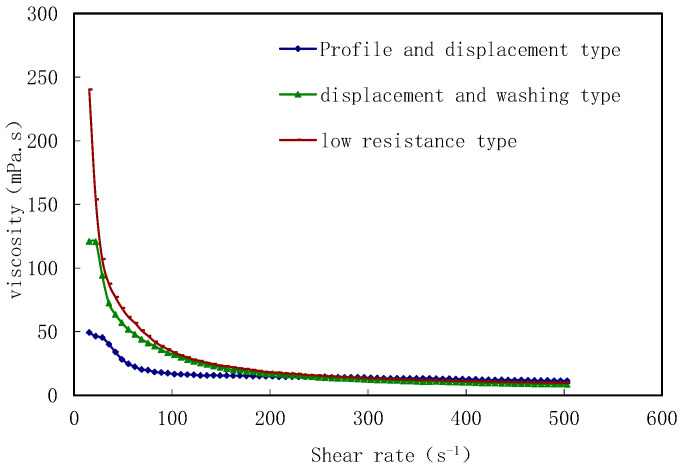
Viscosity curves of different types of polymeric surfactant.

Three types of poly-surfactant solution—profile control and displacement type (PD), displacement and washing type (DW), and low-resistance type (LR)—were prepared at a concentration of 1000 mg/L, and their viscosities were measured at different shear rates. It was observed that the viscosity of all three types of poly-surfactants decreased with an increase in shear rate, classifying them as shear-thinning fluids (Figure 1).

#### 2.1.3. Viscosity Stability

A 1000 mg/L poly-surfactant solution was prepared, and viscosity was tested under anaerobic conditions at 45 °C for different durations. The viscosity values for the three types of polymeric surfactants noticeably increased with prolonged storage time. Additionally, the higher the concentration, the larger the increased range, and the 60-day viscosity retention rate of the poly-surfactants exceeded 100% (Table 1).

#### 2.1.4. Salt Resistance

As the NaCl concentration increased, the polymeric surfactant agent exhibited a significant increase in viscosity (Table 2). The robust salt resistance of the polymer surface agent enabled the maintenance of high viscosity when prepared with wastewater. This capability not only conserves polymer dosage but also enhances economic benefits.

#### 2.1.5. High Viscoelasticity

**Figure 2:**
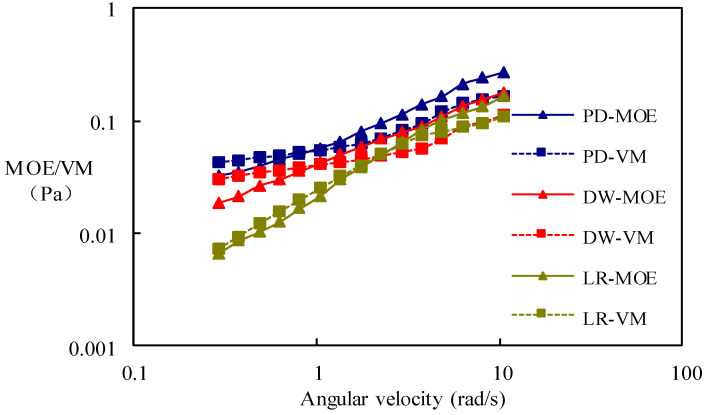
MOE/VM with angular velocity of different poly-surfactants.

Figure 2 illustrates the variation curves of the modulus of elasticity (MOE) and viscous modulus (VM) with angular velocity for different polymeric surfactants at a concentration of 1000 mg/L. The MOE represents the elastic modulus, also referred to as the energy storage modulus, reflecting the amount of energy stored by the material during elastic deformation, indicating the material’s elasticity. On the other hand, the VM is also known as the energy dissipation modulus, reflecting the amount of energy lost due to thermal deformation caused by viscous deformation during material deformation, thereby indicating the material’s viscosity. Analyzing the curve, it is evident that all three types of polymeric surfactant exhibit high viscoelasticity.

### 2.2. The Influencing Factors of Oil Displacement Effect

#### 2.2.1. Viscosity

In the artificial core measuring 10 cm × 2.5 cm in diameter, water flooding was conducted until a water cut of 98% was achieved (in oil well production, the water cut, representing the water cut in the produced liquid, reflects the well’s oil production capacity). Subsequently, a poly-surfactant injection of 0.57 PV (PV represents the pore volume, indicating the ratio of injection volume to core pore volume and reflecting the magnitude of injection volume) was carried out, with a 30% water-releasing rate of emulsion. (Poly-surfactants exhibit a strong emulsifying effect on crude oil, forming a mixed liquid. If water and oil separation occurs too rapidly, the carrying capacity for crude oil decreases, affecting the oil recovery efficiency.) Following the poly-surfactant injection, water flooding was resumed until a water cut of 98% was again attained. Table 3 demonstrates that the viscosity, ranging between 40 mPa·s and 70 mPa·s, corresponds to better oil displacement, with a total EOR of 73% achieved when the viscosity is 60 mPa·s. (EOR is a critical parameter for evaluating the current development level of oil fields. In the experiment, the oil volume is initially recorded by saturating the core, and subsequently, the displaced crude oil volume is recorded through an oil displacement test. The recovery efficiency is obtained by dividing these two volumes. Therefore, the recovery rate represents the proportion of the oil produced to the total crude oil, expressed as a percentage.)

#### 2.2.2. Dehydrating Rate of Emulsion

Based on the results from Section 3.2.1, we selected a viscosity of 70 mPa·s for the emulsion test, keeping all other conditions consistent with those in Section 2.2.1, except for the water-releasing rate. The examined data presented in Table 4 reveal that the oil displacement effect is optimal when the water-releasing rate of the emulsion falls within the range from 30% to 70%. Specifically, when the water-releasing rate is at 50%, the total EOR reaches 71.2%, indicating the most favorable outcome.

#### 2.2.3. Permeability

Building upon the viscosity selection of 70 mPa·s from Section 2.2.2, a 30% water-releasing rate of emulsion was chosen for testing, maintaining all other conditions consistent with those in Section 3.2.2. Examining the data presented in Table 5 reveals a noticeable decrease in the EOR value with decreasing permeability. The displacement effect is most pronounced when effective permeability is above 400 mD (Table 5). Specifically, the EOR of poly-surfactants can reach 15% or more under these conditions.

### 2.3. Study on the Mechanism of Micro-Oil Displacement by Polymeric Surfactant Flooding

The primary mechanism of EOR through poly-surfactant flooding is to enhance oil displacement efficiency through emulsification and flow rate control. Oil can be separated from emulsions by some methods such as high-voltage electric fields, chemical demulsification, centrifugation, and vacuum separation. Vacuum separation is a pure physical separation method that has been widely used. In the experiment, a photolithographic glass core was used to simulate real pore conditions. The glass core’s pore was initially filled with crude oil, followed by water flooding until a water cut of 98% was achieved. Subsequently, the poly-surfactant was injected with a volume of 0.8 PV. Considering the relatively small injection volume, the effect was not explicitly noticeable; the displacement time for the poly-surfactant was adjusted to 2 h, equivalent to injecting a volume of polymer surfactant at 2.0 PV. The process was observed through video recording, concluding with water flooding until a water cut of 98% was once again attained (Table 5).

#### 2.3.1. Enhanced Oil Recovery

Observations reveal that the oil recovery of the PD polymeric surfactant consistently surpassed that of DW at the same displacement time. Additionally, the oil recovery demonstrated an upward trend with the increase in displacement time. Notably, when the recovery degree was low during the water flooding period, subsequent polymeric surfactant flooding yielded higher recovery efficiency. The analysis of viscosity, elasticity, and interfacial tension of the polymeric surfactant indicated that PD poly-surfactant exhibited higher viscosity and elasticity compared to DW, while the interfacial tension of 5.5 × 10^−3^ mN/m of PD was lower than that of DW (6.4 × 10^−3^ mN/m). This observation aligns with the conclusion that low-interfacial-tension and high-viscoelastic-displacement fluid results in a higher recovery value (Table 6).

#### 2.3.2. Mechanism of Micro-Oil Displacement

##### Poly-Surfactant Flood Expands Sweep Volume

**Figure 3:**
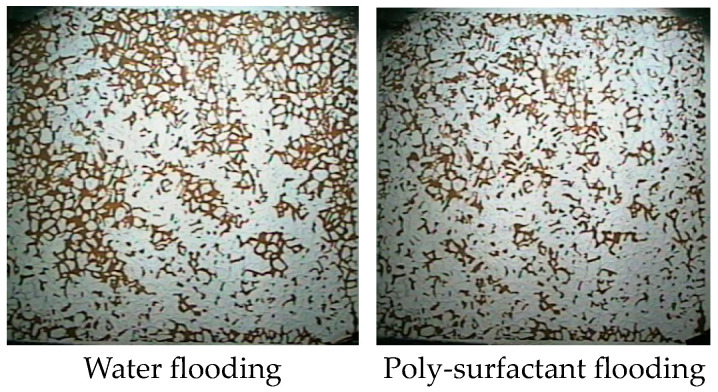
Residual oil distribution after water and poly-surfactant flooding.

The higher viscosity of poly-surfactants contributes to an expanded oil sweep area, enhancing recovery efficiency under the condition that the injection rate of the polymeric surface must not be higher than that of water injection. This effect is evident in Figure 3, where residual oil after poly-surfactant flooding is noticeably lower than in water-flooded scenarios.

##### Poly-Surfactant Flood Drives the Residual Oil

**Figure 4:**
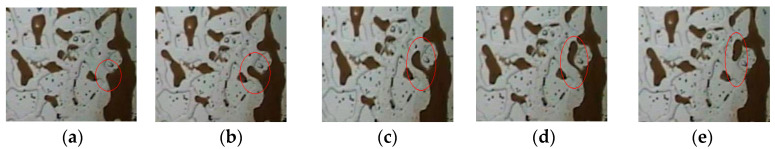
The large areas of residual oil were pulled and cut. (**a**) residual oil film pulled up (**b**) pulled into a columnar shape (**c**) further stretching (**d**) forming small columnar oil (**e**) aggregation with movable oil.

To clearly observe the impact of poly-surfactants on residual oil after water flooding, the analysis focuses on the area at the entrance of the core through a video recording. Figure 4 includes images taken from video recordings, illustrating that the poly-surfactants effectively displaced residual oil in the column, cluster, film, and blind areas after water flooding.

**Figure 5:**
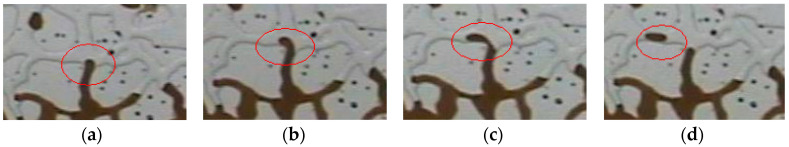
The remaining oil is pulled into oil droplets. (**a**) columnar residual oil (**b**)the remaining oil in the column is pulled (**c**) being stretched and cut (**d**) forming movable oil droplets.

During poly-surfactant flooding (Figure 5), a substantial expanse of residual oil is extracted and segmented into large fluid droplets. This phenomenon is attributed to the significantly higher interfacial viscosity between poly-surfactants and oil compared to that of water and oil. Additionally, the shear stress of poly-surfactants and oil surpasses that of water and oil. In this scenario, the tension exerted on the extensive oil, in the direction of shear motion, imparts the poly-surfactant solution with the ability to transport oil.

**Figure 6:**
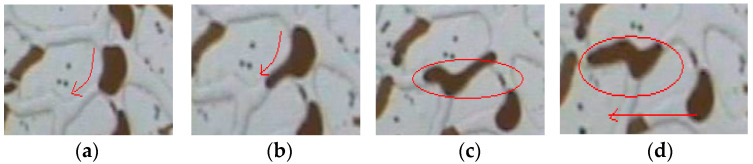
Deformational flow of residual oil droplets through a narrow channel. (**a**) oil droplets in pores (**b**) being driven into the narrow channel (**c**) transforming into channel (**d**) accumulation form large oil droplets.

**Figure 7:**
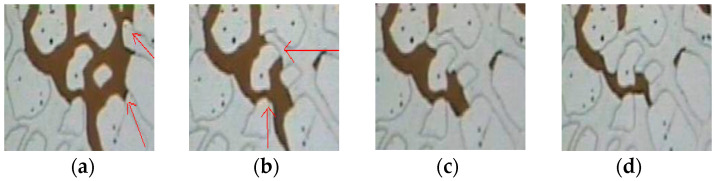
Starting and displacement process of cluster oil by polymeric surfactant. (**a**) remaining oil in pores (**b**) clustered residual oil is displaced along the arrow direction (**c**) clustered residual oil is gradually displaced from the core (**d**) the remaining oil has been effectively displaced.

The flow process of the large oil drop through the narrow channel is observed as the large oil drop traverses the narrow channel. Under the influence of the viscoelasticity of the poly-surfactant and the reduction in interfacial tension, the oil droplets initially deform into a small channel. Subsequently, they form a fine columnar oil structure through the channel (Figure 6 and Figure 7). During the flow through the porous medium, a normal stress perpendicular to the displacement direction is generated by the solution containing a viscoelastic poly-surfactant. This viscoelastic surfactant solution exhibits the ability to pull the oil flow from behind and the sides.

## 3. Materials and Methods

### 3.1. Materials

**Figure 8:**
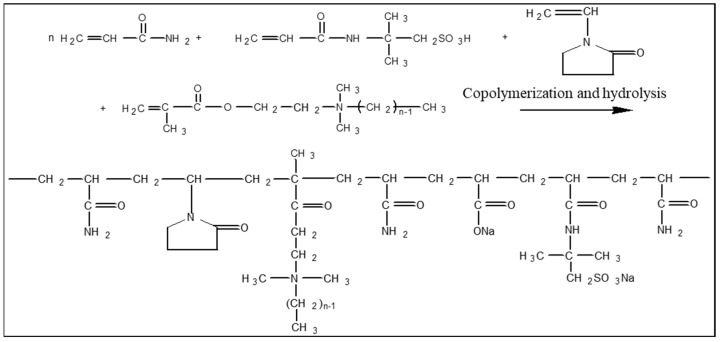
Molecular formula of polymeric surfactant.

There are three common types of poly-surfactant that are used in the Daqing oil field (Figure 8): the profile control and displacement type (PD), the displacement and washing type (DW), and the low-resistance type (LR). A PD poly-surfactant is primarily employed for profile control and blocking higher permeability layers, while DW-type poly-surfactants play a major role in emulsification and oil flooding, making them widely used. The water and oil used in the experiments were sourced from the 1st oil production plant of the Daqing oil field. For the experiments, natural small sandstone columnar cores with dimensions of 2.5 cm (diameter) × 10 cm (length) were used, and the permeability of the cores ranged from 350 to 500 mD. Rock permeability measurements were conducted based on Darcy’s law. By measuring the pressure difference at both ends of the core sample and the flow rate through the sample, permeability could be calculated using the corresponding Darcy formula, considering the viscosity of the fluid used.

In the core oil displacement experiment, after the core was loaded, it was necessary to pressurize the annular space of the core loader until the annular pressure reached about 10 MPa and let it remain still for a period of time. If the annular pressure remained unchanged or was guaranteed to be above 9 MPa, it could be used.

The core holder was a three-axis core gripper. We applied different axial and radial pressures to the end face and surrounding cylindrical surfaces of the rock sample, which are mostly used for displacement testing.

The crude oil used in the experiments was extracted from the oil production plant, and its viscosity was measured to be 9.8 mPa·s under formation temperature. The experimental water was an effluent diluted with pure water, and its salinity was 6800 mg/L.

### 3.2. Instrumentation

#### 3.2.1. Viscosity

**Figure 9:**
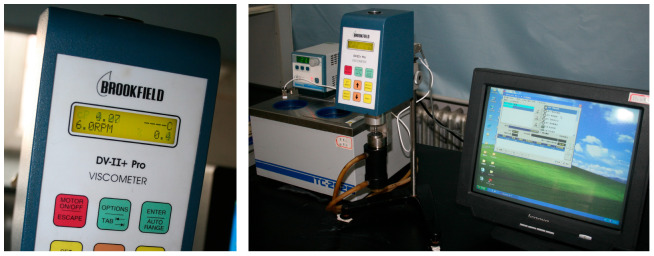
Brookfield viscometer display.

Viscosity measurements were conducted using a Brookfield LVDV-II+Pro (Figure 9). The unique molecular structure of polymeric surfactants can lead to significant fluctuations in viscosity values. To address this issue, a computer program was employed to control the Brookfield viscometer. This allowed for continuous detection over a specified period (300 s) under the set temperature, speed, and other conditions. The viscometer obtained viscosity values every second, and the final output comprised the viscosity value and calculation of the average viscosity. This approach improves the reliability of the data and reduces the random error associated with the measurements.

#### 3.2.2. Flow Characteristics 

The test equipment used for studying flow characteristics comprised an advection pump, a core holding unit, pressure sensors, intermediate containers, a hand pump, and pipeline gates. All of these components, except for the advection pump and hand pump, were placed in a 45 °C incubator to maintain consistent temperature conditions during the experimentation.

#### 3.2.3. Oil Displacement Test

(1) Select rock cores for vacuum pumping; to ensure the effectiveness, vacuum pumping should be carried out for at least 2 h. (2) Calculate the porosity and permeability of the water measurement model using a saline solution with a salinity of 6800 configured for saturated simulated groundwater. (3) Saturate oil at 45 °C and let it stand for 1–2 days after saturation to simulate formation aging. (4) According to the experimental plan, simulate the actual displacement rate of the oil layer by first injecting water to a water cut of 98%. (5) Inject the polymeric surfactant solution (0.57 PV) and then water to a water cut of 98%. (6) Replace the rock core and repeat steps (1) to (5).

#### 3.2.4. Micro-Oil Displacement Test

(1) Vacuum the microscopic model and saturate the oil. (2) Simulate the displacement rate of the oil layer by water until the model does not produce oil. (3) Inject a polymer surfactant solution at a constant rate, observe the residual oil condition after oil displacement, and record dynamic images during the displacement process. (4) Analyze the image and calculate the oil displacement efficiency under this displacement condition. (5) Change the polymeric surfactant system according to the experimental sequence and repeat steps (2) to (4). (6) Clean the rock core and end the experiment.

## 4. Conclusions

Poly-surfactant agents exhibit a notable property of increasing viscosity. Their displacement effect is most pronounced when core effective permeability exceeds 400 mD, the viscosity falls within the range of 40–70 mPa·s, and the water-releasing rate of emulsion is maintained between 30% and 70%. The higher viscosity of poly-surfactants contributes to an expanded oil sweep area, enhancing recovery efficiency and resulting in significantly less residual oil compared to water flooding. During poly-surfactant flooding, a substantial amount of residual oil is extracted and transformed into large fluid droplets, emphasizing its effectiveness in oil displacement. The application prospects of polymeric surfactants are promising due to their high emulsion-carrying capacity and high EOR; however, the difficulty of oil–water separation in the produced fluids still restricts their widespread use in oil fields.

## Figures and Tables

**Table 1:** Viscosity stability data of polymeric surfactants made with wastewater.

Sample Name	Concentration mg/L	Viscosity (mPa·s)							
		0 d	1 d	3 d	7 d	15 d	30 d	45 d	60 d
PD type	1200	454	737	724	683	747	767	695	680
DW type	1200	206	248	288	356	365	271	245	241
LR type	1200	114	134	163	178	219	143	112	125

**Table 2:** Data for salt resistance of polymeric surfactants.

Sample Name	NaCl Concentration (mg/L)						
	1000	3000	4000	5000	6000	7000	9000
PD viscosity	141	214	370	527	561	558	464
DW viscosity	139	153	192	181	180	150	136
LR viscosity	89	60	55	59	65	66	55

**Table 3:** Effect of viscosity on oil displacement effect.

Core Number	Viscosity (mPa·s)	Effective Permeability (mD)	Oil Saturation (%)	EOR of Water Flood (%)	EOR of Poly-Surfactant (%)	Total EOR (%)
1	40	425.29	68.41	53.19	17.02	70.21
2	60	444.95	68.38	53.76	19.36	73.12
3	70	422.72	68.49	52.63	17.90	70.53
4	100	445.19	69.11	53.13	15.10	68.23
5	130	419.50	68.69	53.68	14.21	67.89
6	150	470.70	69.68	53.61	13.81	67.42

**Table 4:** Effect of water evolution rate on oil displacement effect.

Core Number	Water Evolution Rate (%)	Effective Permeability (mD)	Oil Saturation (%)	EOR of Water Flood (%)	EOR of Poly-Surfactant (%)	Total EOR (%)
1	0	444.73	68.99	53.16	11.26	64.42
2	10	468.68	69.23	52.82	14.75	67.57
3	30	446.70	69.06	53.20	17.10	70.30
4	50	444.87	68.84	53.05	18.17	71.22
5	70	467.49	69.07	52.71	15.14	67.85
6	90	445.31	68.89	52.63	12.95	65.58

**Table 5:** Permeability effect on oil displacement effect.

Core Number	Effective Permeability (mD)	Oil Saturation (%)	EOR of Water Flood (%)	EOR of Poly-Surfactant (%)	Total EOR (%)
1	231.01	66.20	46.71	11.51	58.22
2	478.04	68.00	48.52	15.62	64.14
3	722.03	70.30	51.01	17.53	68.54

**Table 6:** The recovery factor of PD and DW displacement with different times.

Poly-Surfactant Type	Water Flooding Time	Water Flood EOR (%)	Poly-Surfactant Time	Injection Pore Volume (PV)	EOR in 50 min (%)	EOR in 90 min (%)	EOR in 120 min (%)	Total EOR (%)
DW	3 h 20 min	42.73	2 h	2.0	10.36		12.36	55.09
PD	3 h 20 min	44.89	2 h	2.0	16.53		17.75	62.64
DW	40 min	35.75	90 min	0.8	17.44	18.65		54.40
PD	40 min	36.47	90 min	0.8	18.74	20.08		56.55

## Data Availability

The economic parameters and well production parameters in this paper were all obtained in the Daqing oil field. They are currently in use and considered reliable.

## Abbreviations

EOR	Enhanced oil recovery
ASP	Alkali/surfactant/polymer
PD	Profile control and displacement-type polymeric surfactant
DW	Displacement and washing-type polymeric surfactant
LR	Low-resistance-type polymeric surfactant
PV	Pore volume
MOE	Modulus of elasticity
VM	Viscous modulus
